# Protective Vaccination Reshapes Hepatic Response to Blood-Stage Malaria of Genes Preferentially Expressed by NK Cells

**DOI:** 10.3390/vaccines8040677

**Published:** 2020-11-13

**Authors:** Marcos J. Araúzo-Bravo, Denis Delic, Daniela Gerovska, Frank Wunderlich

**Affiliations:** 1 Group of Computational Biology and Systems Biomedicine, Biodonostia Health Research Institute, 20014 San Sebastián, Spain; daniela.gerovska@biodonostia.org; 2 IKERBASQUE, Basque Foundation for Science, 48009 Bilbao, Spain; 3 TransBioNet Thematic Network of Excellence for Transitional Bioinformatics, Barcelona Supercomputing Center, 08034 Barcelona, Spain; 4 Boeringer Ingelheim Pharma, 88400 Biberach, Germany; 5 Department of Biology, Heinrich-Heine-University, 40225 Düsseldorf, Germany; Frank.Wunderlich@hhu.de

**Keywords:** liver-resident NK (lrNK) cells, conventional NK (cNK) cells, protective vaccination, blood-stage malaria, natural killer gene complex (NKC), NKC-localized NK cell receptors

## Abstract

The role of natural killer (NK) cells in the liver as first-line *post infectionem* (*p.i.*) effectors against blood-stage malaria and their responsiveness to protective vaccination is poorly understood. Here, we investigate the effect of vaccination on NK cell-associated genes induced in the liver by blood-stage malaria of *Plasmodium chabaudi.* Female Balb/c mice were vaccinated at weeks 3 and 1 before being infected with 10^6^ *P. chabaudi*-parasitized erythrocytes. Genes preferentially expressed by NK cells were investigated in livers of vaccination-protected and non-protected mice on days 0, 1, 4, 8, and 11 *p.i.* using microarrays, qRT-PCR, and chromosome landscape analysis. Blood-stage malaria induces expression of specific genes in the liver at different phases of infection, i.e., *Itga1* in expanding liver-resident NK (lrNK) cells, *Itga2* in immigrating conventional NK (cNK) cells; *Eomes* and *Tbx21* encoding transcription factors; *Ncr1, Tnfsf10, Prf1, Gzma, Gzmb, Gzmc, Gzmm,* and *Gzmk* encoding cytolytic effectors; natural killer gene complex (NKC)-localized genes encoding the NK cell receptors KLRG1, KLRK1, KLRAs1, 2, 5, 7, KLRD1, KLRC1, KLRC3, as well as the three receptors KLRB1A, KLRB1C, KLRB1F and their potential ligands CLEC2D and CLEC2I. Vaccination enhances this malaria-induced expression of genes, but impairs *Gzmm* expression, accelerates decline of *Tnfsf10* and *Clec2d* expression, whereas it accelerates increased expression of *Clec2i*, taking a very similar time course as that of genes encoding plasma membrane proteins of erythroblasts, whose malaria-induced extramedullary generation in the liver is known to be accelerated by vaccination. Collectively, vaccination reshapes the response of the liver NK cell compartment to blood-stage malaria. Particularly, the malaria-induced expansion of lrNK cells peaking on day 4 *p.i.* is highly significantly (*p* < 0.0001) reduced by enhanced immigration of peripheral cNK cells, and KLRB1F:CLEC2I interactions between NK cells and erythroid cells facilitate extramedullary erythroblastosis in the liver, thus critically contributing to vaccination-induced survival of otherwise lethal blood-stage malaria of *P. chabaudi*.

## 1. Introduction

Malaria is still a major threat of human health in tropical countries, causing globally an estimated 228 million cases and 405,000 deaths in 2018, in particular about 266,000 deaths in children aged under 5 years [1]. An effective anti-malaria vaccine is not yet commercially available [2,3]. However, promising vaccine candidates are being tested. The most prominent candidate, RTS,S, has been recently reported to induce partial protection, which, however, is obviously dependent on age of vaccines and wanes with time [2].

Malaria is caused by parasitic protozoans of the genus *Plasmodium*. Only their blood-stages are responsible for morbidity and mortality, which develop and multiply within host red blood cells [3]. The deadliest human malaria species is *Plasmodium falciparum*, causing about 99% of global malaria-related deaths [1]. The main effector organ against blood-stage malaria is the spleen, which is able to eliminate senescent and other aberrant erythrocytes including *Plasmodium*-parasitized erythrocytes [4,5]. Additionally, the liver with its intrinsic immune system is increasingly recognized as an effector organ against blood-stage malaria, though the liver is known for its tolerogenic milieu [6]. In particular, Kupffer cells with their erythrophagocytic capacity are currently regarded as mainly responsible for removal of *Plasmodium*-infected erythrocytes in the liver [7,8,9,10]. Moreover, there is increasing information, though controversially debated, that natural killer (NK) cells in the liver may also play a critical role in the elimination of *Plasmodium*-infected erythrocytes [11,12]. Indeed, their innate cytotoxic activity makes NK cells an important player in the first-line defense against *Plasmodium*-infected erythrocytes long before activation and development of adaptive immune mechanisms [12,13,14]. Nevertheless, NK cells may be even capable of shaping subsequent adaptive immune mechanisms and also possess memory-like adaptive immune features [13,14,15,16,17].

NK cells encompass heterogeneous cell populations and are currently considered as a special subset of group 1 innate lymphoid cells (ILC1s) [11,12,18]. Their common function is to kill diseased cells such as virus-infected, tumor, and stressed cells by releasing perforin and granzymes from their cytoplasmic vesicles via exocytosis, which induce apoptosis of the targeted cells. Humans possess 5 different granzymes, whereas mice exhibit a larger repertoire of 10 granzymes (A, B, C, D, E, F, G, K, M, and N), whose encoding genes are clustered among three different chromosomes, i.e., the GzmA cluster on chromosome 13, the GzmB cluster on chromosome 14, and the GzmM cluster on chromosome 10 [19,20]. However, it is not yet known whether all these 10 *Gzm*s are activated in response to blood-stage malaria in the liver and whether protective vaccination affects expression of malaria-responsive *Gzm*s in the liver at all.

A plethora of germline-encoded killer cell lectin-binding receptors (KLRs) on the surface of NK cells recognize diseased cells and ultimately activate or inhibit the cytolytic activities of NK cells by either a “missing-self mechanism”, e.g., deficient in MHC class I expression, or an “induced-self” mechanism [21,22]. Many of the genes encoding KLRs are localized within the natural killer gene complex (NKC) on chromosome 12 in humans and on chromosome 6 in mice [23,24,25]. The NKC in mice contains three major gene clusters. At the distal end, genes of the *Ly49* (*Klra*) cluster encode receptors of homodimeric type II glycoproteins of the C-type lectin-like superfamily. Incidentally, the KLRA/LY49 receptors are functionally comparable, though structurally totally different, to the killer cell immunoglobulin-like receptors (KIR) in humans [26]. Centromeric from the *Klra* cluster, the bifunctional *Cd94/Nkg2* (*Klrd1/Klrc*) gene cluster is localized [27,28]. At the proximal end, genes of the third *Nkrp1* (*Klrb1*) cluster encode the KLRB1 receptor family, whose members are evolutionary related to the KLRA/LY49 family, but they do not survey MHC class I ligands [23,24,29]. Instead, they bind to various members of the C-type-lectin-related receptors (CLR; previously termed OCIL; current nomenclature: CLEC2) encoded by genes of the *Clec2* family, which are localized in the same subregion of the NKC as the *Klrb1* gene cluster [24,25]. At present, however, there exists only rudimentary knowledge on which members of the different *Klr* and *Clec2* gene families are expressed in the liver, which are responsive to blood-stage malaria, and which can be affected by vaccination—if at all.

Heterogeneity of NK cells is not only defined by their expression pattern of surface KLRs and cytolytic effectors, but also by their tissue distribution. The liver, for example, is currently thought to contain predominantly liver-resident NK (lrNK) cells, which in turn differ from tissue-resident NK (trNK) cells in other organs [30,31,32]. The lrNK cells are presumably derived from liver-resident progenitor cells and are therefore thought to represent a distinct lineage of NK cells, which are different from circulating conventional NK (cNK) cells. The cNK cells are characterized by surface expression of CD49b and the transcription factors eomesodermin (Eomes) and T-bet, while the lrNK cells express only T-bet, TRAIL, and CD49a (for reviews see [12] and [33]). However, it has not been investigated yet whether the relative proportions of lrNK and cNK cells in the liver are affected by vaccination and/or blood-stage malaria, respectively.

Features of NK cells in the liver in response to vaccination and blood-stage malaria can be studied experimentally only in animal models, as, e.g., *Plasmodium chabaudi* in mice, which shares several characteristics with *P. falciparum* [34,35]. In the *P. chabaudi* model, vaccination with an experimental non-infectious vaccine induces a healing course of otherwise non-healing lethal blood-stage infections [36,37]. Primary infections with 10^6^ *P. chabaudi*-parasitized erythrocytes take a similar time course in the surviving vaccinated Balb/c mice as in non-vaccinated mice, with the difference that non-vaccinated mice exhibit a higher peak parasitaemia on approximately day 8 *post infectionem* (*p.i.*) and succumb to infection after peak parasitaemia during crisis, whereas over 80% of the vaccinated mice survive the infections [37].

Vaccination-induced survival is associated with significantly altered responses of the liver to blood-stage malaria at different phases of infections as shown previously [38,39,40,41,42,43]. Most remarkably, vaccination was found to accelerate extramedullary erythroblastosis in the liver induced by blood-stage infections of *P. chabaudi* [44]. This has been experimentally evidenced as significantly accelerated expression of erythroid genes in the liver, in particular those genes encoding constituents of erythrocyte surface membranes. As a continuation of these previous studies, here we aim to study systematically the possible effects of vaccination on gene expression associated with the NK cell compartment of the liver of mice infected with blood-stage malaria of *P. chabaudi*. Specifically, gene expression microarray technology, quantitative RT-PCR, and chromosome landscape analysis are used to identify and validate malaria- and vaccination-responsive genes known to be preferentially expressed by NK cells during the course of *P. chabaudi* blood-stage infections with a lethal outcome in non-vaccinated mice and a healing outcome in vaccination-protected mice.

## 2. Materials and Methods

### 2.1. Mice

Only female Balb/c mice aged 10–12 weeks were used throughout the experiments. They were obtained from the central animal facilities of the University of Düsseldorf, where they were bred under specified pathogen-free conditions. Mice received standard diet (Wohrlin, Bad Salzuflen, Germany) and water ad libitum throughout the experiments.

### 2.2. Protective Vaccination

Mice were vaccinated with a non-infectious vaccine under identical experimental conditions as described elsewhere [37,44]. The vaccine consisted of erythrocyte ghosts isolated from *P. chabaudi*-parasitized erythrocytes as detailed previously [36,45]. These membrane ghosts were previously characterized to contain parasite-synthesized proteins and presumably autoantigens [46,47]. Approximately 10^6^ ghosts suspended in 100 µl Freund’s complete adjuvant (FCA) were subcutaneously injected at weeks 3 and 1 before challenge with blood-stages of *P. chabaudi* malaria. In parallel, control mice were treated with only FCA. Protective efficacy of the vaccination procedure depends on genes of the H-2 complex and the non-H-2 background, as well as on sex and testosterone of mice. For instance, only one vaccination is sufficient to protect over 90% of female B10.A mice with “malaria-self-healer” non-H-2 background and “malaria-non-healer” H-2 haplotype, whereas only about 55% of male mice are protected and only about 34% of female mice when pretreated with testosterone for 4 weeks [48]. However, female Balb/c mice with “malaria-non-healer” non-H-2 background and “malaria-selfhealer” H2^d^-haplotype [49], as used here, require two-time vaccination to raise the survival rates [37].

### 2.3. Blood-Stage Malaria of P. chabaudi

*P. chabaudi* infections were maintained in outbred mice by weekly passages of infected blood under sterile conditions. The non-clonal line of *P. chabaudi,* used in our laboratory since 1982 [50], resembles *P. chabaudi* AS in terms of restriction fragment length polymorphism as well as dihydrofolate reductase and cysteine protease sequence identities [51]. Moreover, the used line of *P. chabaudi* is self-healing as the AS clone, which is under control of genes of the H-2 complex and the non-H-2 background as well as sex and sex hormones of the infected mouse strain [49]. The Balb/c mice were challenged with approximately 10^6^ *P. chabaudi*-infected erythrocytes. Parasitaemia was evaluated in Giemsa-stained blood smears and erythrocytes were counted in a Neubauer chamber as described previously [37].

### 2.4. Liver Sampling During Primary Blood-Stage Infections

Both vaccinated (V) and non-vaccinated (N) mice were concomitantly infected with 10^6^ *P. chabaudi*-parasitized erythrocytes at one week after the last vaccination. These primary infections take a similar course in terms of parasitaemia in vaccinated and non-vaccinated mice. The prepatent phase of infections lasts for about 3 days, before the patent period begins on day 4 *p.i.* with the appearance of 1–5% parasitized erythrocytes in the peripheral blood without any difference between vaccinated and non-vaccinated mice. Peak parasitaemia is reached on approximately day 8 *p.i.*, with about 60% in non-vaccinated mice and only about 40% in vaccinated mice. The following crisis phase, lasting for approximately 3–4 days, is characterized by dramatically falling parasitaemias to 5–1% and the death of all non-vaccinated mice, whereas over 80% of the vaccinated mice survive the infections [37]. For liver sampling, groups of 3 animals from both vaccinated and non-vaccinated mice were sacrificed at different phases of infections: upon infection on day 0 *p.i*., at early pre-patency on day 1 *p.i.*, at early patency on day 4 *p.i*., at peak parasitaemia on day 8 *p.i*., and towards the end of the crisis phase on day 11 *p.i*. Parasitaemia was previously determined in blood smears of individual mice sacrificed in the different groups [41], which corresponded to that determined in living infected mice under identical experimental conditions [37]. Livers were aseptically removed from sacrificed mice, rapidly frozen in liquid nitrogen, and stored at −80°C until use. Both groups of vaccinated and non-vaccinated mice contained 4 mice, which were not sacrificed for liver sampling. All 4 mice in the non-vaccinated group succumbed to infection during crisis, whereas 3 mice in the vaccinated group survived the infection for at least 3 weeks, in accordance with previous results [37].

### 2.5. Hybridization of Mouse Whole Genome Oligo Microarrays

These experiments were also previously performed and their results recently published elsewhere [44]. In brief, total RNA from aliquots of individual frozen livers “pulverized” under liquid nitrogen was isolated by the standard Trizol protocol (Qiagen, Hilden, Germany) followed by cleaning with the miRNeasy Kit (Qiagen). Equivalents of 100 ng from each RNA sample were taken to produce Cy3-labeled cRNA using the Agilent Low Input Quick Amp Labeling Kit (Agilent Technologies) according to the manufacturer’s protocol. Agilent’s 8 × 60 K oligo microarrays (design number 028005) were used for hybridization with the Agilent Gene Expression Hybridization Kit.

### 2.6. Analyses of Microarrays

The gene expression microarrays were scanned with the Agilent’s Microarray Scanner System (Agilent Technologies). The Agilent Feature Extraction (FE) software was used to read out and process the microarray image files. FE software determines feature intensities (including background subtraction), rejects outliers, and calculates statistical confidences of feature intensities. Microarrays were normalized over all 30 samples by the quantile method. Global transcriptomics analyses over all normalized 30 microarrays were performed including a heat map of the most highly variable transcripts, hierarchical clustering dendrograms (calculated using the unweighted pair group method with arithmetic mean and Euclidean distance measure), and Principal Component Analysis (PCA) [44]. Microarray data are available at both the EMBL-EBI Array Express repository (Array accession number: E-MTAB.6494) and the NCBI’s Gene Expression Omnibus (GEO) database with accession number GSE129133 (https://www.ncbi.nlm.nih.gov/geo/query/acc.cgi?acc=GSE129133). Gene expression profiles were determined from the normalized microarrays prepared from the individual livers of both vaccinated and non-vaccinated mice during infections with *P. chabaudi* on days 0, 1, 4, 8, and 11 *p.i*. and are summarized as log_2_-transformed data in the heat map presented in the Supplementary Table S1. Gene expression levels were measured as light intensities above normalization level and were given as means ± standard deviation (SD) as a dispersion metric in all figures. T-test was used to determine the statistical significance of differentially expressed genes (DEGs) between vaccinated and non-vaccinated mice both at a given day *p.i.* and during the intervals between the different sampling days *p.i*.

### 2.7. Selection of DEGs

Statistically significant DEGs in the liver of vaccinated vs. non-vaccinated mice on day 4 *p.i*. were determined by calculating the mean values of each probe across all the samples of each of the two groups. Next, we filtered out all the probes whose absolute value of difference of mean values between the two groups was less than a selection threshold *θ_DEG_* = 1 (that corresponds to a fold change of 2 in log_2_ scale), and we applied the Student’s *t*-test. The multitest effect influence was tackled through control of the False Discovery Rate using the Benjamini-Hochberg method for correcting the initial *p*-values with significance threshold *α_DEG_* = 0.001.

### 2.8. Chromosomal Landscape Analysis of DEGs

To evaluate the chromosomal enrichment of the DEGs between the vaccinated and non-vaccinated populations on day 4 *p.i*., each DEG was mapped onto its corresponding chromosome, and we compared the number of DEGs mapped onto a chromosome with the number of genes on each chromosome. We estimated the statistical significance of the enrichment by the *p*-value calculated using the hypergeometric distribution using a significance threshold. The multitest effect was tackled through control of the False Discovery Rate using the Benjamini-Hochberg method for correcting the initial *p*-values with significance threshold *α_LAN_* = 0.01. To analyze the grouping of the gene *loci* on a chromosome, a one-dimensional clustering technique was applied that searched for groups of gene *loci* located at a distance smaller than *D_clu_* = 2,000,000 bps, and that considered them as a cluster if they were populated by a minimum number *N_min_* = 4 of genes [52].

### 2.9. Quantitative real-time PCR

High-Capacity cDNA Reverse Transcription Kit (Life Technologies) and TaqMan mRNA assays (Life Technologies) were used to perform reverse transcription of mRNAs coding for the following proteins: ITGA1 (assay ID: Mm01306375_m1), ITGA2 (assay ID: Mm00434371_m1), KLRG1 (assay ID: Mm00516879_m1), GZMC (assay ID: Mm01313651_m1), GZMN (assay ID: Mm00461851_m1), KLRD1 (assay ID: Mm00495182_m1), KLRB1F (assay ID: Mm04211785_m1), and CLEC2I (assay ID: Mm00777071_g1). PCR reactions were carried out with the TaqMan^®^ gene expression master mix (Life Technologies) according to the instructions given by the manufacturer on a 7900HT real-time PCR System, as previously described [44]. Raw Ct values were calculated using the SDS software v.2.4 with GAPDH (assay ID: Mm99999915_g1) for normalization. Fold change of expression was calculated with the comparative Ct method (2-ΔΔCt) [53]. Data sets were analyzed for statistical significance using two-tailed unpaired heteroskedastic Student’s *t*-test (* = *p*<0.05).

## 3. Results

### 3.1. Vaccination Reshapes the Response to Blood-Stage Malaria of Genes Expressed by lrNK and cNK Cells in the Liver

**Figure 1:**
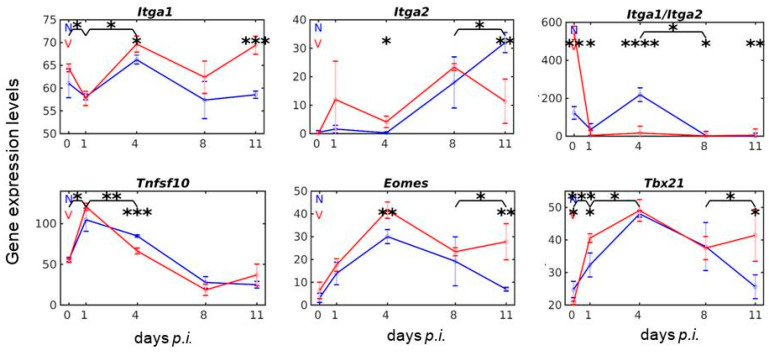
Time courses of hepatic expression of genes associated with liver-resistant natural killer (lrNK) cells and conventional natural killer (cNK) cells. RNA was isolated from individual livers prepared from vaccination-protected (V, red) and non-vaccinated (N, blue) mice infected with *Plasmodium chabaudi* blood-stage malaria on days 0, 1, 4, 8, and 11 *p.i*. Gene expression levels in linear scale are plotted as the means of three microarrays ± SD. The number of “*” marks over interval lines between sampling points and over the sampling points indicate statistical significance of corresponding intervals and of given sampling points, respectively, between vaccinated and non-vaccinated mice: * *p* < 0.05; ** *p* < 0.01; *** *p* < 0.001; **** *p* < 0.0001.

Liver-resident NK cells are known to express the integrin alpha1 subunit (CD49a) encoded by *Itga1* on their surface, while cNK cells express the *Itga2*-encoded integrin alpha2 subunit (CD49b) [30,31,32]. Figure 1 shows the time course of expression of *Itga1* and *Itga2* in the liver of both vaccination-protected mice and non-vaccinated mice infected with *P. chabaudi*. Conspicuously, both genes respond to malaria and vaccination, though differently in several aspects. First, constitutive expression of *Itga1* on day 0 *p.i.* is much higher with approximately 64 above normalization level than that of *Itga2* with approximately 0.16. Secondly, expression of *Itga1* in response to malaria takes a totally different course in comparison with that of *Itga2*. There is a slight dampening of *Itga1* expression during early pre-patency between day 0 and 1 *p.i*., before expression increases, reaching the first maximum with approximately 67 already at early patency on day 4 *p.i.* Thereafter, expression decreases to approximately 58 at peak parasitaemia on day 8 *p.i*. and remains at that level until the end of the crisis phase on day 11 *p.i*. (Figure 1). By contrast, the malaria-induced expression of *Itga2* is impaired for at least four days, before expression begins to linearly increase on day 4 *p.i.,* reaching its maximum with approximately 32 above normalization level only shortly before death of non-vaccinated mice on day 11 *p.i*. (Figure 1). Remarkably, this maximal expression level of 32 for *Itga2* is still significantly lower than that of *Itga1* with approximately 58 at the same time-point. Thirdly, vaccination induces significant changes in the course of the malaria-induced expressions of both *Itga1* and *Itga2* in the liver. Expression of *Itga1* is significantly decreased from approximately 64 to 58 during early pre-patency and then increases to maximum expression of approximately 69 on day 4 *p.i*., which is significantly higher than that in non-vaccinated mice. Thereafter, *Itga1* expression in vaccinated mice declines as in non-vaccinated mice, before it begins again to increase at peak parasitaemia on day 8 *p.i*. reaching a second maximum of approximately 69 on day 11 *p.i*. (Figure 1). By contrast to *Itga1*, vaccination induces a slight, but not significant, increase in expression of *Itga2* on day 1 *p.i*., followed by a decline reaching a level of approximately 5 on day 4 *p.i.,* which is significantly higher than that in non-vaccinated mice at the same time. Then, *Itga2* expression takes a sharp increase as that in non-vaccinated mice, but reaches a maximum of approximately 24 already at peak parasitaemia, before declining to approximately 13 above normalization level towards the end of the crisis phase on day 11 *p.i.,* which is significantly (*p* < 0.001) lower than that of *Itga1* with 69 in non-vaccinated mice (Figure 1). Obviously, there appears to exist a reciprocal relationship in vaccinated mice between *Itga1* transcript levels and those encoded by *Itga2*, i.e., increased expressions of *Itga2* coincide with decreased expressions of *Itga1* and vice versa. Fourthly, Figure 1 also shows the ratio of *Itga1* vs. *Itga2* over infection in both non-vaccinated and vaccinated mice. The most prominent effect is that the malaria-induced peak on day 4 *p.i*. in non-vaccinated mice is largely absent in vaccination-protected mice at a very high significance (*p* < 0.0001). This indicates that protective vaccination affects the malaria-induced changes in NK cell composition of the liver in mice. Obviously, the liver of vaccination-protected female Balb/c mice, not infected with blood-stage malaria of *P. chabaudi*, contains a much higher proportion of lrNK cells than cNK cells, evidenced by a high ratio of constitutive expression of *Itga1* vs. *Itga2*. Upon infection with blood-stage malaria, however, this ratio of lrNK cells to cNK cells is decreased, significantly more in vaccinated mice than in non-vaccinated mice, presumably due to an increase in the relative proportion of cNK cells vs. lrNK cells (Figure 1).

LrNK cells have been described to express the surface ligand TRAIL encoded by *Tnfsf10* and cNK cells the transcription factor eomesodermin encoded by *Eomes*, while the transcription factor T-bet encoded by *Tbx21* is expressed in both cNK cells and lrNK cells [12,33]. Figure 1 shows that *Tnfsf10* reveals a high constitutive expression, which is in the range of that of *Itga1*. Totally different to *Itga1*, however, *Tnfsf10* reveals an enormous increase in expression upon infection with malaria between day 0 and 1 *p.i*., followed by a significantly declined expression reaching its minimum on day 8 *p.i.* and 11 *p.i.* in non-vaccinated mice. In vaccinated mice, however, the early increase in expression of *Tnfsf10* is still significantly higher and the subsequent declining expression is even accelerated, i.e., it is highly significantly (*p* < 0.001) lower on day 4 *p.i*. than in non-vaccinated mice (Figure 1). Figure 1 also shows the course of expression of *Eomes* and *Tbx21* in the liver of vaccinated and non-vaccinated mice in response to malaria. In non-vaccinated mice, both genes show similar time courses of expression with a maximal expression on day 4 *p.i*., which is significantly lower for *Eomes*, whereas *Tbx21* exhibits a higher constitutive expression. Additionally, vaccinated mice reveal maximal expression of Eomes and *Tbx21* on day 4 *p.i*., however, with the difference that maximal expression of *Eomes* is significantly higher in vaccinated mice than in non-vaccinated mice and that *Tbx21* reveals a significantly stronger increase in expression in vaccinated than in non-vaccinated mice very early between day 0 *p.i*. and day 1 *p.i*. Both *Tbx21* and *Eomes* exhibit a significantly increased expression between day 8 and 11 *p.i*. (Figure 1).

### 3.2. Vaccination Reshapes Malaria-Induced Expression of Ncr1, Prf1 and Gzm Genes in the Liver

**Figure 2:**
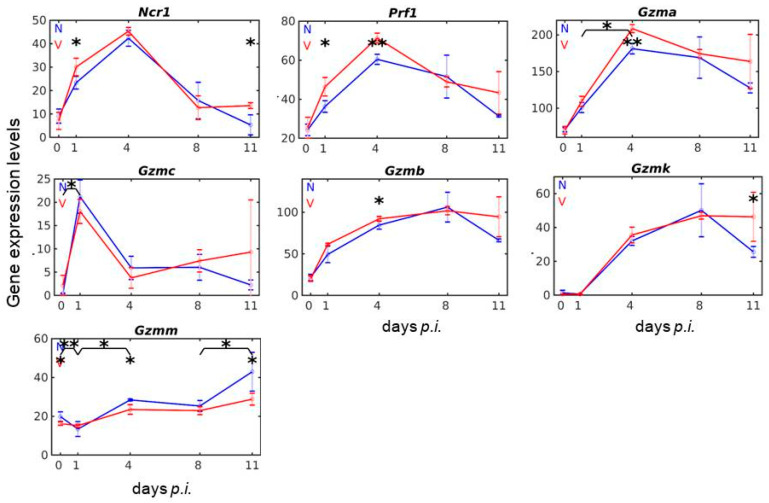
Expression trajectories of genes encoding cytolytic effector molecules in the liver of vaccinated and non-vaccinated mice. RNA was isolated from individual livers prepared from vaccination-protected (V, red) and non-vaccinated (N, blue) mice infected with *P. chabaudi* blood-stage malaria on days 0, 1, 4, 8, and 11 *p.i.* Gene expression levels in linear scale are plotted as the means of three microarrays ± SD. The number of “*” marks over interval lines between two sampling points and over the sampling points indicate statistical significance of corresponding intervals and of given sampling points, respectively, between vaccinated and non-vaccinated mice: * *p* < 0.05; ** *p* < 0.01.

The microarrays used contain oligo probes for the natural cytotoxicity triggering receptor 1 (*Ncr1*), for perforin (*Prf1*), and for all 10 members of the granzyme (*Gzm*) gene family. The profile time series in Figure 2 shows that blood-stage infections with *P. chabaudi* malaria induce the expression of *Ncr1* in the liver, which reaches its maximum with approximately 39 above normalization level on day 4 *p.i*., before it declines to such a low level on day 11 *p.i*., which is typical for constitutive expression on day 0 *p.i*. In contrast to non-vaccinated mice, however, vaccination-protected mice respond to infection with a significantly higher expression of *Ncr1* on day 1 *p.i*. and on day 11 *p.i*. Figure 2 also shows that blood-stage malaria in non-vaccinated mice induces an increase in hepatic mRNA levels of *Prf1* with a maximum at early patency on day 4 *p.i*. Vaccination-protected mice reveal significantly higher expressions of *Prf1*, not only at maximum expression on day 4 *p.i*., but also earlier on day 1 *p.i*.

Among the 10 members of the Gzm family, there are five members, i.e., *Gzmd, Gzme, Gzmf, Gzmg*, and *Gzmn*, which respond neither to malaria nor to vaccination. One member, namely *Gzmc*, is responsive to malaria with an early maximum mRNA expression on day 1 *p.i*., and this malaria-induced early increase in *Gmzc* expression appears to be responsive to vaccination (Figure 2). The other four members of the *Gzm* gene family, namely *Gzma, Gzmb, Gzmk*, and *Gzmm*, also significantly respond to both malaria and vaccination. Figure 2 shows the time series of expressions of these four *Gzm* genes during lethal blood-stage infections in non-vaccinated mice and healing infections in vaccination-protected mice. The expression courses differ among the four *Gzms* both in vaccinated and non-vaccinated mice. The expression courses of *Gzma* in both vaccinated and non-vaccinated mice are most similar to those taken by *Prf1* with a maximum on day 4 *p.i*. *Gzmb* expression increases strongest between day 0 and day 1 *p.i*. and reaches its maximum at peak parasitaemia on day 8 *p.i.* This maximal expression is maintained in vaccinated mice until day 11 *p.i*., whereas there is a decline in non-vaccinated mice. The expression of *Gzmk* takes a similar course as *Gzmb* both in non-vaccinated and vaccinated mice on days 4, 8, and 11 *p.i*., whereas the expression between day 0 and day 1 *p.i*. is totally impaired. The expression response of *Gzmm* to malaria and vaccination differs from that of the other three Gzms in as far as maximal expression occurs only on day 11 *p.i*., and vaccinated mice exhibit significantly lower expressions on day 4 *p.i*. and day 11 *p.i.* than in non-vaccinated (Figure 2).

### 3.3. Enrichment of NKC with Up-Regulated DEGs on Chromosome 6 in Vaccinated Mice on Day 4 p.i.

**Figure 3:**
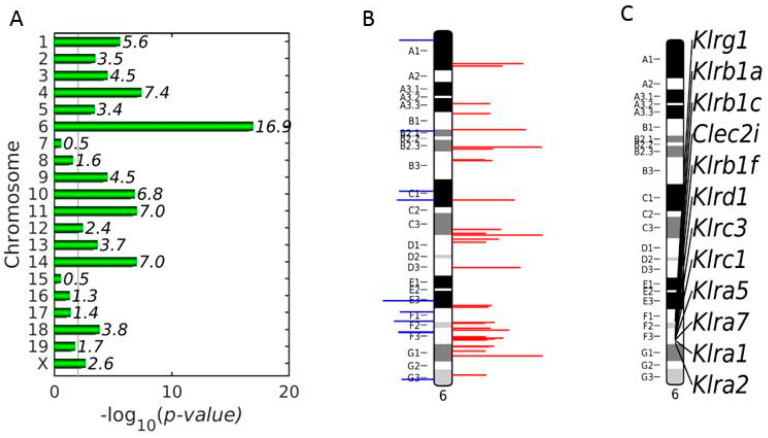
Chromosome landscape analysis of the differentially expressed genes (DEGs) in the liver between vaccinated and non-vaccinated mice on day 4 *p.i.* (**A**)–log_10_(*p*-value) of the enrichment of each chromosome with up-regulated DEGs in the vaccinated population in relation to the non-vaccinated one on day 4 *p.i*. The most enriched chromosome is the chromosome 6. (**B**) Ideogram of chromosome 6 with all DEGs in the vaccinated population in relation to the non-vaccinated one on day 4 *p.i*. The horizontal blue and red lines mark the *loci* of the down- and up-regulated DEGs, respectively, and their lengths are proportional to the average level of expression. (**C**) Ideogram of chromosome 6 with the uni-dimensional clustering of the up-regulated DEGs in vaccinated mice in relation to non-vaccinated mice on day 4 *p.i*. The natural killer cell complex (NKC) is localized in the F3 cytoband and its up-regulated DEGs are shown on the right.

To search for chromosomes statistically enriched with up-regulated DEGs in the liver of vaccinated mice in relation to non-vaccinated mice, a chromosome landscape analysis was performed at early patency on day 4 *p.i*., when *P. chabaudi*-infected erythrocytes begin to appear in peripheral blood of mice. Figure 3A shows that chromosome 6 is the most highly statistically significantly enriched (*p* = 1.26 × 10^−17^) with DEGs, whose expression is up-regulated in vaccination-protected mice. Visual inspection of the ideogram of chromosome 6 with both up- and down-regulated DEGs point out the existence of potential clusters of up-regulated DEGs (Figure 3B). To identify these clusters, we performed one-dimensional clustering that searched for groups of gene *loci* of the up-regulated genes [52]. Four clusters were identified to be distributed in cytobands C3-D1, F2, F3, and G1. The cluster of cytoband F3 is entirely populated with genes encoding proteins of the NKC (Figure 3C).

### 3.4. Vaccination Reshapes Malaria-Responsive Expression of Klra Genes in the Liver

**Figure 4:**
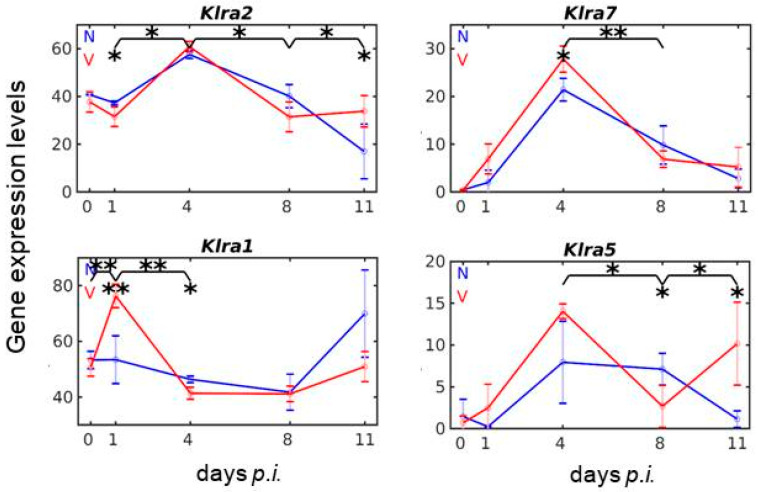
Expression trajectories of genes encoding members of the KLRA receptor family involved in MHCIa-dependent tissue surveillance. RNA was isolated from individual livers prepared from vaccination-protected (V, red) and non-vaccinated (N, blue) mice infected with *P. chabaudi* blood-stage malaria on days 0, 1, 4, 8, and 11 *p.i.* Gene expression levels in linear scale are plotted as the means of three microarrays ± SD. The number of “*” marks over interval lines between two sampling points and over the sampling points, respectively, indicate statistical significance of two corresponding intervals and of a given sampling point, respectively, between vaccinated and non-vaccinated mice: * *p* < 0.05; ** *p* < 0.01.

The *Ly49/Klra* gene family is highly polymorphic and its gene content varies among mouse strains [22,23,24,25]. The *Ly49/Klra* haplotype of Balb/c mice used in the present study is obviously the smallest among the four mouse strains hitherto analyzed. It contains the six framework genes *Ly49q/Klra17, Ly49e/Klra5, Ly49i/Klra9, Ly49g/Klra7, Ly49c/Klra3,* and *Ly49a/Klra1,* all of them encoding inhibitory receptors [22,25,54]. *Ly49l/Klra12*, localized between *Klra3* and *Klra7*, is the only gene encoding an activating receptor [22,55]. *Klra17* is described to be expressed only on myeloid cells [56]. All these *Klra* genes, except *Klra3*, are represented in the used microarrays by at least one oligo probe and are found to be expressed in the liver of both vaccinated and non-vaccinated mice at varying levels and on different days *p.i*. (Figure 4).

By far the lowest expression among all *Klra* genes in response to malaria is exhibited by the activating receptor-encoding *Klra12,* whose mRNA level is only minimal above the normalization level in the liver of non-vaccinated mice. This minimal level is not significantly affected by vaccination. A much higher expression than *Klra12* displays *Klra9,* but its malaria-response is not affected by vaccination. By contrast, expressions of *Klra2, Klra7, Klra1, and Klra5* are responsive to malaria and are significantly changed in vaccination-protected mice (Figure 4). Vaccination induces significantly increased expressions of *Klra2* between days 1 and 4 *p.i*. and between days 8 and 11 *p.i*., as well as decreased expressions between day 4 and 8 *p.i*. (Figure 4). Additionally, *Klra7* responds to both malaria and vaccination, with a maximal expression level on day 4 *p.i.*, which is significantly higher in vaccination-protected mice (Figure 4). As with *Klra7,* the expressions of *Klra1* and *Klra5* respond to both malaria and vaccination, albeit differently. *Klra1* expression induced by blood-stage malaria in the liver of non-vaccinated mice reveals a late maximum with a mRNA level of approximately 70 above normalization level towards the end of the crisis phase on day 11 *p.i*., while in vaccination-protected mice, a very early maximum expression of approximately 78 occurs at early pre-patency on day 1 *p.i.* before declining to a minimum of approximately 40 at early patency on day 4 *p.i.* (Figure 4). The expression of *Klra5* is significantly higher on day 4 *p.i*. and day 11 *p.i.* in the liver of vaccinated mice than in non-vaccinated mice (Figure 4).

### 3.5. Vaccination Reshapes Malaria-Induced Expression of Cd94/Nkg2 Genes

**Figure 5:**
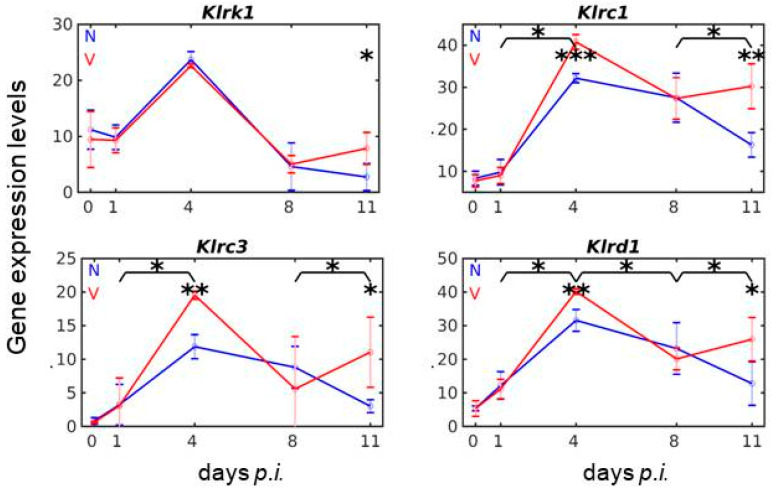
Time series of NK-expressed genes encoding KLR receptors involved in MHCIb-dependent tissue surveillance. RNA was isolated from individual livers prepared from vaccination-protected (V, red) and non-vaccinated (N, blue) mice infected with *P. chabaudi* blood-stage malaria on days 0, 1, 4, 8, and 11 *p.i*. Gene expression levels in linear scale are plotted as the means of three microarrays ± SD. The number of “*” marks over interval lines between two sampling points and over a given sampling point indicate statistical significance between two corresponding intervals and between a given sampling point, respectively, between vaccinated and non-vaccinated mice: * *p* < 0.05; ** *p* < 0.01; *** *p* < 0.001.

Centromeric to the *Klra* subregion of the NKC is localized the *Cd94/Nkg2* complex with the genes *Klrd1(Cd94), Klrk1 (Nkg2d), Klrc3 (Nkg2e), Klrc2 (Nkg2c2), and Klrc1 (Nkg2a)* [24,25]. The three receptors KLRC1–3 form obligate disulfide-bonded heterodimers with KLRD1, while the unrelated KLRK1 forms homodimers [25]. The used microarrays contain probes for all five genes. *Klrc2* is the only gene that exhibits a very low constitutive expression and responds neither to malaria nor to vaccination. The other four genes are both malaria- and vaccination-responsive, as shown in Figure 5. *Klrd1, Klrc1*, and *Klrc3* reveal similar expression courses in response to malaria and to vaccination (Figure 5). Maximum expression of all three genes occurs on day 4 *p.i.* in both vaccinated and non-vaccinated mice, but the maximum is always significantly higher in the liver of vaccination-protected mice. Additionally, vaccination-protected mice reveal a significantly higher increase in expression of all three genes on day 11 *p.i*. than non-vaccinated mice (Figure 5).

The unrelated *Klrk1* exhibits its expression maximum of approximately 24 above normalization level on day 4 *p.i.* in both non-vaccinated and vaccinated mice. Only towards the end of crisis on day 11 *p.i*., there is a higher expression of *Klrk1* in the liver of vaccinated mice than in non-vaccinated mice. The activating receptor KLRK1 is known to be a master regulator of immune cell responsiveness and to affect all stages of the life cycle of NK cells through modification of NK cell receptor activation thresholds, which plays a role in effector responses on NK cells in the periphery [56,57].

### 3.6. Vaccination Reshapes Responsiveness of Klrb1/Nkrp1 Gene Expression to Malaria

**Figure 6:**
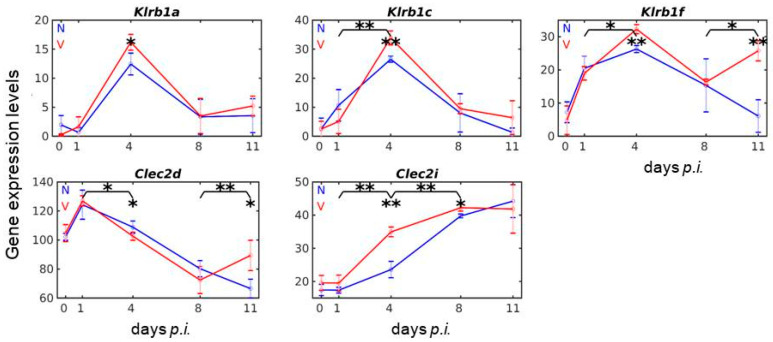
Time series of expressions of *Klrb1* and *Clec2* genes encoding receptors and ligands involved in MHCI-independent tissue surveillance. RNA was isolated from individual livers prepared from vaccination-protected (V, red) and non-vaccinated (N, blue) mice infected with *P. chabaudi* blood-stage malaria on days 0, 1, 4, 8, and 11 *p.i*. Gene expression levels in linear scale are plotted as the means of three microarrays ± SD. The number of “*” marks over interval lines between two sampling points and over sampling points indicate statistical significance between two corresponding intervals and between a given sampling point, respectively, between vaccinated and non-vaccinated mice: * *p* < 0.05; ** *p* < 0.01.

The *Klrb1* cluster of the NKC, localized centromeric from the *Cd94/Nkg2* subregion [25], contains the genes *Klrb1b (Nkrp1b/d)* and *Klrb1g (Nkrp1g*) encoding inhibitory receptors, and *Klrb1a (Nkrp1a), Klrb1c (Nkrp1c),* and *Klrb1f (Nkrp1f)* coding for activating receptors, with *Klrb1a* and *Klrb1c* as orphan-activating receptors with unknown physiological ligands [24,58,59]. The used microarrays do not contain any oligo probes for *Klrb1g.* The probe for *Klrb1d* is not detecting any significant amounts of corresponding mRNA in the liver of vaccinated and non-vaccinated mice infected with blood-stage malaria. *Klrb1b,* which is represented by three different probes on the microarray, also does not respond to malaria and to vaccination, as it has been expected, since *Klrb1b* is expressed as NK1.1 in C57BL/6 mice, but not in Balb/c mice used in the present study. The other three genes, i.e., *Klrb1a, Klrb1c*, and *Klrb1f*, respond both to malaria and to vaccination (Figure 6). The malaria-induced *Klrb1a* and *Klrb1c* genes are responsive to vaccination, evidenced by a highly significant up-regulation of their mRNA levels on day 4 *p.i.* The vaccination-responsiveness of *Klrb1f* is evidenced by highly significantly up-regulated mRNA levels at early patency on day 4 *p.i*. and towards the end of the crisis phase on day 11 *p.i.* (Figure 6).

### 3.7. Vaccination reshapes malaria-induced expression of Clec2 genes in the liver

The *Clec2-*encoded C-type-lectin-receptors (CLR) are not only localized in the same NKC-subregion as the *Klrb1 genes* but may even function as ligands for *Klrb1*-encoded receptors [25]. For instance, the *Klrb1d-*encoded receptors recognize *Clec2d-*encoded ligands and *Klrb1f*-encoded receptors ligate with *Clec2i*-encoded ligands [58,59,60]. The used microarrays contain oligo probes for the seven functional *Clec2* genes. Among these, the genes *Clec2f* and *Clec2g* are neither responsive to malaria nor to vaccination, whereas the genes *Clec2e, Clec2h,* and *Clec2j* are responsive to malaria, but not to vaccination. Only the genes *Clec2d* and *Clec2i* are responsive both to malaria and vaccination, though totally differently. Malaria induces increased expressions of *Clec2d* early on day 1 *p.i.,* before expressions decline to lower levels towards the end of the crisis phase on day 11 *p.i*. (Figure 6). Vaccination, however, accelerates this malaria-induced decline of *Clec2d,* but significantly turns this decline to an increased expression towards the end of the crisis phase (Figure 6). Totally different to *Clec2d,* blood-stage malaria impairs early expression of *Clec2i* between day 0 and day 1 *p.i.*, before inducing a continuous increase in expression, reaching a maximum on day 11 *p.i*. in non-vaccinated mice (Figure 6). In vaccination-protected mice, however, the increased expression of *Clec2i* is significantly accelerated on day 4 *p.i.* and reaches its maximum already at peak parasitaemia on day 8 *p.i*. (Figure 6).

### 3.8. Vaccination Reshapes Malaria-Responsive Expression of Cd69 and Klrg1 in the Liver

**Figure 7:**
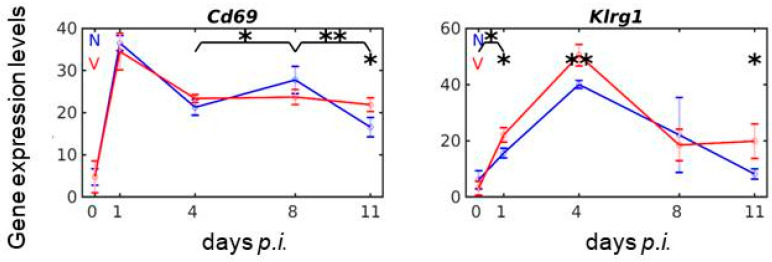
Expression trajectories of *Cd69* and *Klrg1* in the liver of vaccination-protected and non-protected mice. RNA was isolated from individual livers prepared from vaccination-protected (V, red) and non-vaccinated (N, blue) mice infected with *P. chabaudi* blood-stage malaria on days 0, 1, 4, 8, and 11 *p.i*. Gene expression levels in linear scale are plotted as the means of three microarrays ± SD. The number of “*” marks over interval lines connecting two sampling points and over sampling points indicate statistical significance of corresponding intervals and of a given sampling point, respectively, between vaccinated and non-vaccinated mice: * *p* < 0.05; ** *p* < 0.01.

Within the NKC, *Cd69* is localized between *Klrd1/Cd94* and *Clec2d* [25] and encodes an activating receptor known to sustain activation of NK cell cytotoxicity, blockable by KLRD1 [61,62]. However, *Cd69* is also expressed in most of the other haematopoietic cell lineages [63]. Figure 7 shows the profile time series of *Cd69* expression in response to malaria and vaccination. Malaria induces a dramatic increase in *Cd69* expression from approximately 5 to 36 above normalization level very early during day 0 *p.i*. and 1 *p.i*., before declining to approximately 22 on day 4 *p.i.* This early peak in *Cd69* expression is not affected by vaccination, which, however, significantly modulates the ensuing course of the malaria-induced expression of *Cd69* between days 4 and 11 *p.i*. (Figure 7).

*Klrg1* is localized at the proximal part of the NKC, centromeric from the *Klrb1*/*Clec2* complex [24]. It presumably encodes an inhibitory receptor, whose ligands are R- and N-cadherin and, in particular, E-cadherin. Figure 7 shows the malaria- and vaccination-response of *Klrg1* expression. It is obviously the only gene of the NKC whose malaria-induced expression was identified to be responsive to vaccination on three different days *p.i*. during infection, namely on days 1, 4, 11 *p.i*. (Figure 7). In accordance, KLRG1 is known to sustain activation, proliferation, and expansion of NK cells in the liver [64,65].

### 3.9. Validation by Quantitative qRT-PCR of Microarrays Results

**Figure 8:**
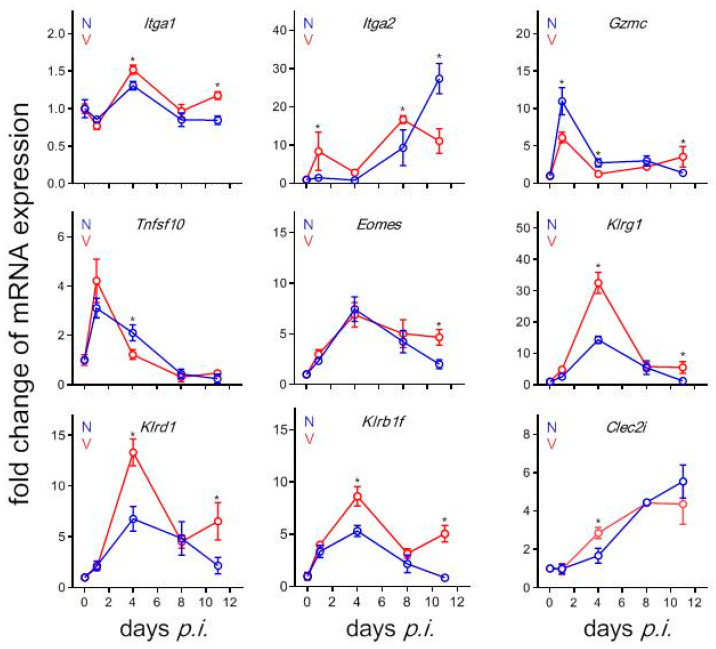
qRT-PCR of mRNAs in the liver of vaccinated (V, red) and non-vaccinated (N, blue) Balb/c mice. RNA was isolated from individual livers prepared from mice infected with *P. chabaudi* on different days p.i. Means ± SD of duplicate determinations performed with liver aliquots from three different mice. “*” indicate significant differences between vaccinated and non-vaccinated mice (*p* < 0.05).

Figure 8 summarizes the qRT-PCR results of the time courses of expression of some selected NK cell-associated genes, whose expression was identified by microarrays to respond both to *P. chabaudi* blood-stage malaria and to vaccination. Expressions of *Itga1* and *Itga2* induced in the liver in response to infection with blood-stage malaria and vaccination take similar courses as those detected by microarrays (cf. Figure 1). Note that the qRT-PCR results are plotted as opposite -fold change expressed mRNA, while the microarray results indicate mRNA levels above the normalization level. In particular, the qRT-PCR data show that *Itga1* expression in response to malaria reveals a maximum in non-vaccinated mice on day 4 *p.i*., while the malaria-induced *Itga2* expression is largely impaired until day 4 *p.i*., before it continuously increases, reaching maximum on day 11 *p.i*. (Figure 8). However, vaccinated mice exhibit an undulatory reciprocal increase of *Itga1* and *Itga2* expression during infections. Furthermore, the expressions of *Tnfsf10* and *Eomes* in response to malaria and vaccination are similar as with those determined by microarrays (cf. Figure 8 with Figure 1). Moreover, similar time course of expressions are detected, both by qRT-PCR and microarrays, for *Klrg1* and *Klrd1* as well as *Klrb1f* and *Clec2i* in both vaccinated and non-vaccinated mice in response to malaria and vaccination (Figure 8).

## 4. Discussion

This study provides evidence that protective vaccination reshapes the response of the liver NK cell compartment to primary blood-stage infections of *P. chabaudi* malaria. Most remarkably, this reshaping encompasses a regrouping of lrNK and cNK cells. Indeed, the liver of mice responds to blood-stage malaria with an increase in the expression of *Itga1* encoding the integrin alpha 1 subunit of CD49a and, also, an increase, but significantly impaired, in the expression of *Itga2* encoding the integrin alpha2 subunit of CD49b. Provided the currently existing consensus that CD49a is exclusively expressed by lrNK cells and CD49b exclusively by cNK cells, the same is also valid for the expression of their corresponding mRNAs. Then, our data indicate a malaria-induced expansion of lrNK cells within the liver, reaching its maximum at early patency on day 4 *p.i.,* which thereafter declines, due to an increased immigration of cNK cells into the liver. Protective vaccination, however, reshapes this malaria-induced regrouping of lrNK and cNK cells. Specifically, vaccination leads to an undulatory but reciprocal increase in both lrNK cells and cNK cells, ultimately resulting in an increase in the relative proportions of both lrNK cells and cNK cells in the liver, with the proportion of cNK cells apparently not exceeding that of lrNK cells. This vaccination-induced regrouping of the NK cell populations highly significantly (*p* < 0.0001) reduces the malaria-induced increase in the proportion of lrNK cells vs. cNK cells occurring in non-vaccinated mice peaking at early patency on day 4 *p.i..* In accordance, previous research reported that *P. chabaudi* malaria is associated with a rapid expansion of NK cells in peripheral blood of “malaria-self-healer” C57BL/6 mice during the pre-patency phase of infection [66]. Further circumstantial evidence comes from vaccination trials with humans using the RTS,S vaccine that caused a disappearance of the transcriptomics signature of peripheral NK cells from the blood [67], interpreted to be due to an immigration of peripheral NK cells “into tissues, possibly the liver” [68].

Protective vaccination was also found to reshape the malaria-induced response of the cytotoxic potency of the liver. The liver responds to malaria with increased expressions of genes encoding cytolytic effector molecules, though differently at different phases of the blood-stage infections, evidenced by increased transcript levels of *Ncr1, Prf1,* and some members of the *Gzm* gene family, i.e., *Gzma, Gzmb, Gzmc, Gzmk,* and *Gzmm*. Significantly enhanced transcript levels by vaccination were found for *Ncr1, Prf1,* and *Gzmc* at early pre-patency on day 1 *p.i.,* for *Prf1, Gzma,* and *Gzmb* at early patency on day 4 *p.i.*, and for *Ncr1* and *Gzmk* towards the end of the crisis phase on day 11 *p.i*.. This increase in transcript levels might be due to the vaccination-induced regrouping and increase in cytotoxic cells in the liver. Anyway, it cannot be unequivocally ascribed to expansion of the more tolerogenic lrNK cells, and/or to immigration of the more cytotoxic peripheral cNK cells, and/or to increases in other cytotoxic non-NK cells. *Gzmm* is the only gene whose malaria-induced up-regulated expression is significantly impaired by vaccination on day 4 *p.i.* and 11 *p.i*. *Gzmm* expression is known to be apparently not regulated by cytokines, as that of the other *Gzm*s. Moreover, GZMMmay also exert extracellular functions, as, e.g., impairment of blood coagulation by cleaving Von Willebrand factor normally stabilizing coagulation factor VIII [69]. This impairment of blood coagulation, besides the increased cytotoxic potency of the liver, might be presumably of benefit for vaccination-induced survival of otherwise lethal blood-stage malaria.

A peculiar response to malaria and vaccination was found for *Tnfsf10* encoding TRAIL, which is currently considered as specifically expressed by lrNK cells [12,33]. Surface expression of TRAIL was described to be promoted by NCR1 [70]. However, the malaria-induced expression of *Tnfsf10* was found to take a totally different course from that of *Ncr1.* Indeed, vaccination enhances the initial malaria-induced increase in *Tnfsf10* expression and even accelerates the following malaria-induced expression decline between day 1 *p.i.* and 8 *p.i*. The transcript level of *Tnfsf10* is highly significantly (*p* < 0.001) lower on day 4 *p.i.* in vaccination-protected mice than in non-vaccinated mice. On the same day, however, the *Ncr1* transcripts have reached their maximum expression level. Additionally, expression of *Itga1* is significantly increased due to expansion of lrNK cells. It might therefore be suspected that the decline in *Tnfsf10* expression between day 1 and 8 *p.i*. is not due to a decrease in the number of the tolerogenic lrNK cells, but rather the TRAIL-mediated cytotoxic mechanism of lrNK cells might be increasingly diminished, possibly to reduce the known risk of TRAIL to promote liver damage by TRAIL-expressing NK cells [71].

Protective vaccination was also found to reshape the malaria-responsive expression of NKC-localized *Klr* genes, preferentially expressed by NK cells. Conspicuously, this vaccination-induced reshaping does not uniformly encompass all *Klr* genes of the NKC expressed in the liver, but only a minority of select members of the three *Klr* gene families. Highly significant (*p* < 0.001) increases in expressions were found for *Klra1* at early patency on day 1 *p.i.* and for *Klrd1*, *Klrc1*, and *Klrc3* at early patency on day 4 *p.i. Klra1* encodes an inhibitory receptor on NK cells known to be involved in the surveillance of “self-peptides” presented by MHC class Ia molecules, as, e.g., H2K^d^ and H2D^d^ expressed on the surface of cells of Balb/c mice. Less abundantly expressed than MHC class Ia molecules are the MHC class Ib molecules known to be encoded by the H2-Q, H2-T, and H2-M gene families [72]. The most studied MHC Ib molecule is Qa-1, whose surveillance is controlled by NK cells expressing KLR heterodimers encoded by *Klrd1/Klrc1* and *Klrd1/Klrc3*, respectively [27,73,74]. Our findings show that the liver displays a higher prevalence of *Klrc1* transcripts encoding the inhibitory KLRC1 in comparison to Klrc3 transcripts encoding the activating KLRC3, in accordance with previous data [27,74]. It is possible that activating KLRD1/KLRC3 receptors might be controlled by the more prevalent inhibitory KLRD1/KLRC1 receptors. The latter are known to be expressed at higher levels by lrNK cells than by cNK cells, which are known for their increased IFNgamma production and degranulation [75]. Even though the relative proportion of lrNK cells in the liver is diminished by immigrating cNK cells in vaccinated mice, their absolute proportion remains still higher than that of cNK cells. The vaccination-induced dynamic regrouping and increase in the number of lrNK cells and cNK cells in the liver during the course of blood-stage infections reasonably explains the vaccination-enhanced expression of the different KLRs preferentially expressed by NK cells involved in MHC class Ia and class Ib surveillance.

Moreover, protective vaccination was also found to reshape the malaria-induced response of the KLRB1:CLEC2 system operating as an MHC class I-independent surveillance system in the liver. Indeed, vaccination highly significantly enhances the malaria-induced maximal expression of *Klrb1f, Klrb1a,* and *Klrb1c* on day 4 *p.i*. and, concomitantly, accelerates the malaria-induced decrease in *Clec2d* expression and increase in *Clec2i* expression. CLEC2I/Clr-g acts as a physiological ligand for KLRB1F [25], whereas CLEC2D is the physiological ligand for KLRB1B acting as a negative regulator of innate immune responses in C57BL/6 mice [27], but not in the *Klrb1b*-negative Balb/c mice used in the present study. The physiological ligands of KLRB1A and KLRB1C are still unknown [25]. Circumstantial evidence, however, suggests that the latter two receptors, which incidentally display a similar structure in C57BL/6 mice explicable by the slight differences in their amino acid sequences [76], can interact with CLEC2D [77], since both receptors are known to stabilize expression of CLEC2D upon ligating with the viral encoded m12 protein expressed on the surface of cells infected with murine cytomegalus virus (MCMV) [78]. On the other hand, rapid down-regulation of CLEC2D at both the cell surface and steady-state transcript levels of infected, stressed, or malignant target cells were described to result in a higher susceptibility to NK cell cytolysis [79,80]. In this context, it is remarkable that *Clec2d* and *Tnfsf10* encoding TRAIL on lrNK cells display almost the same expression time courses in response to both malaria and vaccination, in particular, a significant vaccination-accelerated decline on day 4 *p.i.* It is possible that TRAIL expression by lrNK cells might not only be stimulated by NCR1 [70], but might also require, directly and/or indirectly, stabilization through CLEC2D, whereas its retraction might lead to diminished expression and functioning of TRAIL on lrNK cells [80].

Furthermore, our data indicate a novel function of the MHC class I-independent KLRB1:CLEC2 surveillance system in the control of extramedullary erythroblastosis in the liver. Under identical experimental conditions as used here, the vaccination-accelerated erythroblastosis was recently identified as accelerated expression of erythroid genes, in particular, genes encoding proteins associated with plasma membranes, as, e.g., *Ermap* (Erythroblast membrane associated protein), *Kel* (Kell blood group antigen), *Rhd* (Rh blood group D antigen), and *Cldn13* (Claudin13) [44]. Their expressions in response to malaria and vaccination take about the same time course as that identified here for *Clec2i.* Expression of *Clec2i* is first impaired between day 0 *p.i*. and day 1 *p.i*. and then begins a continuous increase in response to malaria, reaching maximum on day 11 *p.i*., whereas vaccination even accelerates this malaria-induced *Clec2i* expression, evidenced by a highly significant (*p* < 0.001) higher expression on day 4 *p.i.* in vaccinated than in non-vaccinated mice. This supports the view that the transmembrane CLEC2I protein is expressed as a constituent of plasma membranes of erythroblasts and its interaction with its known receptor KLRB1F on NK cells [25] might protect erythroblasts and their progenitors in the liver. Possibly, CLEC2I might be even retained in plasma membranes of peripheral reticulocytes and erythrocytes originating from liver erythroblasts, thus presumably contributing to their lowered penetrability by *P. chabaudi* parasites in peripheral blood [44,80]. In accordance, CLEC2I/Clr-g expression has been described to occur abundantly in haematopoietic cell lines [81], and it is even identifiable in blood cells in the liver, though only by in situ hybridization, while RT-PCR and microarray analyses were both negative due to removal of blood cells before analyses [29]. It is therefore attractive to speculate that KLRB1F-expressing NK cells, increased by vaccination, ligate with CLEC2I expressed in erythroid cells that might facilitate the vaccination-accelerated extramedullary erythroblastosis in the liver. This in turn might lead to an accelerated generation of peripheral reticulocytes and erythrocytes, respectively, and, ultimately, to an accelerated overcome of malaria-induced anaemia and to a healing outcome of otherwise lethal malaria in vaccination-protected mice. Incidentally, it is noteworthy in this context that the interplay of CLEC2I:KLRB1F is known to co-stimulate T cell proliferation and Il-2 production [82], that in turn might contribute to faster development of adaptive immune mechanisms directed against *P. chabaudi* blood-stage malaria.

## 5. Conclusions

Our data add further support to the view that the liver plays a critical role in the outcome of blood-stage malaria. Indeed, vaccination reshapes the response of the NK cell compartment in the liver to primary blood-stage infections of *P. chabaudi* malaria, particularly evidenced by relative impairment of the malaria-induced expansion of lrNK cells peaking on day 4 *p.i*. due to enhanced immigration of cNK cells into the liver. This regrouping of NK cells is associated with increased cytolytic potency of the liver, possibly due to the increased proportion of more cytotoxic cNK cells vs. the more tolerogenic lrNK cells, and with changes in KLRs on NK cells involved in MHC class Ia- and Ib-dependent and -independent surveillance systems. CLEC2I is presumably a constituent on surface membranes of extramedullary erythroblasts in the liver, and its interaction with KLRB1F in NK cells may protect and facilitate extramedullary erythroblastosis accelerated by vaccination in the liver [44]. The vaccination-induced reshaping of the NK cell response in the liver is suggested to be of benefit for vaccination-induced survival of otherwise lethal blood-stage infections of *P. chabaudi*.

## Supplementary Materials

The following are available online at https://www.mdpi.com/2076-393X/8/4/677/s1, Table S1: Heatmap of hepatic expression profiles of NK cell-associated genes used for analyses in the present study.
