# Supplementary material for: Protective Vaccination Reshapes Hepatic Response to Blood-Stage Malaria of Genes Preferentially Expressed by NK Cells
Source: Vaccines (Basel). 2020 Nov 13;8(4):677. doi: 10.3390/vaccines8040677 (PMC7712122; doi:10.3390/vaccines8040677)
Supplement: Supplementary file 1 [file vaccines-08-00677-s001.pdf]

Research Article

Protective vaccination reshapes hepatic response to blood-stage malaria of genes preferentially expressed by NK cells

Supplementary Information

Marcos J. Araúzo-Bravo[1,2*], Denis Delic[3*], Daniela Gerovska[1], Frank Wunderlich[4]

[1] Group of Computational Biology and Systems Biomedicine, Biodonostia Health Research Institute, San Sebastián, Spain; mararabra@yahoo.co.uk (M. J. A.-B.)

[2] IKERBASQUE, Basque Foundation for Science, Bilbao, Spain

[3] Boeringer Ingelheim Pharma, Biberach, Germany; denis.delic@boehringer-ingelheim.com (D. D.)

[4] Department of Biology, Heinrich-Heine-University, Düsseldorf, Germany; Frank.Wunderlich@hhu.de (F. W.)

*Corresponding authors:

Marcos J. Araúzo-Bravo, [a]Group of Computational Biology and Systems Biomedicine, Biodonostia Health Research Institute, San Sebastián, Spain

[b]IKERBASQUE, Basque Foundation for Science, Bilbao, Spain

E-mail: mararabra@yahoo.co.uk

Tel.: +34943006108

Denis Delic, [c]Boeringer Ingelheim Pharma, Biberach, Germany

E-mail: denis.delic@boehringer-ingelheim.com

Tel.: Tel.: +49735154143839

Keywords: Liver-resident NK (lrNK) cells, conventional NK (cNK) cells, protective vaccination, blood-stage malaria, liver transcriptomics

Table S1. Heatmap of hepatic expression profiles of NK cell-associated genes used for analyses in the present study. RNA was isolated from the individual livers prepared from non-vaccinated and vaccinated mice infected with *P. chabaudi* on days 0, 1, 4, 8, and 11 *p.i.*. The 5 columns on the left represent non-vaccinated control mice (N) and the 5 columns on the right the vaccinated mice (V) at the different days *p.i.* (Nd0 – Nd11 *vs* Vd0 – Vd11). Each vertical column contains three vertical lanes of numbers indicating log_2-transformed expression levels of three different microarrays prepared from three different livers at a given time-point. Gene names are indicated on the left.

0 1 2 3 4 5 6 7 8 9 10 11 12 13 14 15 16 17 18

	Nd0			Nd1			Nd4			Nd8			Nd11			Vd0			Vd1			Vd4			Vd8			Vd11		
Itga1	8.0	7.8	7.6	7.7	7.6	7.6	8.1	8.1	8.2	7.3	7.6	7.8	7.7	7.6	7.7	8.1	8.0	8.0	7.7	7.5	7.6	8.3	8.3	8.5	8.1	8.0	7.6	8.2	8.5	8.3
Itga2	0.3	1.1	0.4	1.5	1.6	0.4	0.8	0.3	0.3	3.0	4.2	5.2	5.8	5.3	5.8	0.3	0.3	0.3	2.9	5.2	0.8	2.1	1.4	2.4	4.7	4.8	5.0	3.7	4.2	1.6
Tnfsf10	7.3	7.7	7.5	9.5	10	10	9.2	9.1	9.3	5.9	5.2	4.6	5.3	4.5	5.2	7.5	7.2	7.4	11	11	10	8.1	8.4	7.9	5.1	3.5	4.2	4.9	6.0	7.1
Eomes	0.9	2.0	2.1	2.9	4.2	4.0	5.8	5.3	5.4	4.3	5.5	3.0	2.7	2.5	2.7	3.1	2.6	1.6	4.1	4.0	4.6	6.3	6.8	6.3	4.9	4.6	5.0	5.0	4.6	6.1
Tbx21	4.7	5.2	5.0	5.4	6.0	5.6	7.0	7.0	6.8	6.3	6.6	5.5	5.2	4.6	5.4	4.6	4.6	4.5	6.3	6.5	6.4	7.1	7.2	6.7	6.3	5.8	6.3	6.7	5.7	6.9
Prf1	4.6	5.2	5.0	5.8	6.3	5.9	7.9	7.6	7.8	7.7	7.5	6.2	5.6	5.6	5.6	5.4	5.3	4.4	6.4	7.0	7.0	8.3	8.6	8.5	7.1	6.8	7.1	7.5	6.1	6.1
Gzma	8.4	8.6	8.2	9.6	10	10	13	13	13	13	13	11	11	11	11	8.7	8.3	8.1	10	10	10	14	14	14	13	13	13	14	11	12
Gzmb	5.0	4.7	4.2	6.2	7.6	7.2	9.5	9.0	9.1	10	10	9.2	8.1	8.0	8.2	4.8	4.1	4.5	7.7	7.9	7.9	9.4	9.6	9.7	10	9.8	10	11	8.6	9.4
Gzmk	1.8	0.8	0.7	0.8	0.9	0.8	5.9	5.6	5.5	7.2	8.0	5.8	5.1	4.7	5.3	0.7	0.8	0.8	0.7	0.9	0.7	6.1	6.2	5.5	6.9	6.7	7.0	7.9	5.9	6.4
Gzmm	4.7	4.2	4.4	4.2	3.2	3.6	5.4	5.3	5.3	4.7	5.3	5.0	6.3	7.4	5.9	4.1	3.9	4.1	3.8	3.8	4.0	5.1	4.9	4.6	5.0	4.6	4.7	5.1	5.3	5.7
Gzmc	0.7	0.7	0.7	4.1	4.9	4.8	2.8	2.6	1.8	2.4	3.0	1.9	1.1	1.8	1.6	2.1	0.7	1.3	4.6	4.0	4.1	2.2	1.1	2.3	2.2	2.9	3.0	4.7	2.0	1.4
Ncr1	2.4	3.1	3.4	4.5	5.1	4.9	6.8	6.3	6.4	4.8	4.0	2.8	2.7	0.7	2.9	3.3	2.4	2.1	5.1	5.8	5.6	6.9	6.7	6.6	3.9	2.6	4.0	3.7	3.9	3.5
Klra2	6.4	6.4	6.4	6.0	6.1	6.1	7.6	7.5	7.7	6.7	6.4	5.9	4.8	1.9	4.9	6.3	5.7	6.4	5.8	5.9	5.2	8.0	7.7	7.8	5.6	5.0	6.2	5.6	5.3	6.4
Klra7	0.7	0.7	0.7	2.2	0.8	0.7	4.8	4.3	4.8	3.8	2.8	2.6	2.2	1.7	0.9	0.6	0.7	0.7	2.9	1.8	3.0	5.4	5.4	5.0	2.9	2.6	2.3	2.6	0.6	2.9
Klra1	7.2	7.1	7.5	6.7	7.9	7.3	6.7	6.9	6.8	5.9	6.8	6.7	8.2	9.3	7.5	7.1	7.3	6.9	8.6	9.0	8.6	6.2	6.5	6.5	6.2	6.6	6.5	7.3	7.4	6.7
Klra5	0.5	0.5	2.0	0.5	0.5	0.5	3.6	2.8	1.8	2.8	2.2	2.9	0.5	1.5	1.0	1.3	0.5	0.5	2.4	0.5	1.2	3.8	3.6	3.9	2.3	0.4	1.6	2.2	3.4	3.8
Klrk1	3.2	2.9	3.9	2.7	3.4	3.3	4.9	4.7	5.0	3.0	2.1	0.7	1.6	0.7	2.3	3.9	2.8	2.3	3.2	3.3	2.6	4.8	4.7	4.8	2.5	2.4	1.8	2.9	2.2	3.2
Klrc1	2.9	3.2	2.6	3.1	2.6	3.6	5.7	5.8	5.6	5.7	5.4	4.6	4.2	3.6	4.3	3.0	2.9	2.5	3.1	3.2	2.6	6.2	6.4	6.5	5.7	4.8	5.1	5.8	4.9	5.7
Klrc3	0.8	1.2	0.7	0.8	2.6	1.6	3.4	3.2	3.7	3.4	3.1	2.3	1.9	1.4	1.9	0.9	0.7	0.8	0.9	0.8	2.8	4.4	4.4	4.5	3.8	1.0	1.1	3.5	2.3	3.9
Klrd1	2.5	2.2	2.3	3.4	2.9	4.1	5.9	5.6	5.3	5.3	5.2	3.8	4.2	2.3	3.9	2.8	1.9	2.1	2.8	3.7	3.4	6.3	6.4	6.3	4.8	4.1	4.6	5.7	4.4	5.2
Klrb1a	1.8	1.5	0.4	0.5	0.4	1.3	3.6	3.7	3.2	2.5	1.9	0.4	2.2	0.5	2.4	0.7	0.4	0.5	1.1	1.9	0.4	3.9	4.2	4.0	2.5	0.4	2.0	2.4	2.5	1.8
Klrb1c	2.6	0.7	0.7	2.1	3.8	3.7	5.2	5.2	5.0	3.6	3.3	0.7	1.8	0.7	0.8	2.4	1.2	0.8	2.5	2.9	0.7	5.8	6.0	5.6	3.3	2.8	3.1	3.5	0.8	2.5
Klrb1f	3.2	1.9	2.8	4.2	5.0	4.4	5.1	5.1	5.2	4.5	4.4	2.5	3.0	0.7	3.0	3.1	1.6	1.5	4.5	4.5	4.1	5.8	5.7	5.5	4.2	4.0	4.0	4.7	5.2	5.3
Clec2d	10	10	10	10	11	11	10	10	10	9.0	8.6	9.2	8.1	7.8	8.6	10	10	10	11	11	11	10	10	10	8.8	7.9	8.8	8.9	9.4	10
Clec2i	4.3	4.3	3.9	4.1	4.3	4.2	5.1	4.8	4.7	6.4	6.3	6.3	6.5	7.0	6.3	4.6	4.1	4.5	4.4	4.1	4.7	6.0	5.8	6.0	6.4	6.5	6.6	7.1	6.1	6.2
Cd69	2.5	2.3	1.6	5.9	6.2	6.1	4.6	4.8	4.4	5.5	5.3	4.9	4.3	3.8	4.1	2.7	0.7	2.6	6.0	6.1	5.4	4.7	4.8	4.9	5.1	4.8	4.7	4.9	4.5	4.6
Klrg1	2.4	3.1	1.8	3.7	4.0	4.1	6.4	6.3	6.2	5.5	5.4	2.6	3.1	2.5	3.0	2.3	0.7	1.9	4.4	4.8	4.9	6.8	7.1	7.4	4.7	3.5	4.6	5.1	3.7	4.4
